# Plant Responses to Abiotic Stresses and Rhizobacterial Biostimulants: Metabolomics and Epigenetics Perspectives

**DOI:** 10.3390/metabo11070457

**Published:** 2021-07-16

**Authors:** Motseoa M. Lephatsi, Vanessa Meyer, Lizelle A. Piater, Ian A. Dubery, Fidele Tugizimana

**Affiliations:** 1 Department of Biochemistry, University of Johannesburg, Auckland Park, Johannesburg 2006, South Africa; motseoalephatsi@gmail.com (M.M.L.); lpiater@uj.ac.za (L.A.P.); idubery@uj.ac.za (I.A.D.); 2 School of Molecular and Cell Biology, University of the Witwatersrand, Private Bag 3, WITS, Johannesburg 2050, South Africa; vanessa.meyer@wits.ac.za; 3 International Research and Development Division, Omnia Group, Ltd., Johannesburg 2021, South Africa

**Keywords:** abiotic stress, plant defences, biostimulants, plant growth-promoting rhizobacteria (PGPR), DNA methylation, histone modifications, epigenetics, metabolomics, priming

## Abstract

In response to abiotic stresses, plants mount comprehensive stress-specific responses which mediate signal transduction cascades, transcription of relevant responsive genes and the accumulation of numerous different stress-specific transcripts and metabolites, as well as coordinated stress-specific biochemical and physiological readjustments. These natural mechanisms employed by plants are however not always sufficient to ensure plant survival under abiotic stress conditions. Biostimulants such as plant growth-promoting rhizobacteria (PGPR) formulation are emerging as novel strategies for improving crop quality, yield and resilience against adverse environmental conditions. However, to successfully formulate these microbial-based biostimulants and design efficient application programs, the understanding of molecular and physiological mechanisms that govern biostimulant-plant interactions is imperatively required. Systems biology approaches, such as metabolomics, can unravel insights on the complex network of plant-PGPR interactions allowing for the identification of molecular targets responsible for improved growth and crop quality. Thus, this review highlights the current models on plant defence responses to abiotic stresses, from perception to the activation of cellular and molecular events. It further highlights the current knowledge on the application of microbial biostimulants and the use of epigenetics and metabolomics approaches to elucidate mechanisms of action of microbial biostimulants.

## 1. Introduction

As sessile organisms, plants are constantly exposed to adverse environmental perturbations, such as abiotic stresses. Anthropogenic contributions and increasing climate change continuously exacerbate the detrimental effects of these stresses on crop productivity, thereby posing a threat to global food security. Evolutionarily, plants have developed a multi-layered, complex and highly regulated immune system and defences that involve sensing various danger signals and integration of this information to produce appropriate responses to diverse challenges, ensuring growth and development [1]. These response mechanisms that are induced by stress exposure result in gene expression reprogramming and phenotypic modifications which, in turn, give rise to acquired memorisation, that can either be transient or long-lasting [2]. As plants are repeatedly exposed to different adverse environmental conditions, it is advantageous for plants to be able to remember past stress occurrences for adaptation and defence. In the last decade, progress has been made in elucidating and describing stress memory mechanisms in plants. One of these systems is known as defence priming, which sensitises and prepares the plant for future abiotic stress conditions [3]. 

Priming or pre-conditioning (of plant defences and adaptive mechanisms) as stress memory is a state in which plants are rendered more resistant to subsequent stresses, displaying faster and more efficient defence responses [4,5,6]. Multiple examples of stress memory in response to stimuli such as drought, salinity and cold in higher plants have been shown across several species and discussed in great detail [7,8]. In this regard, numerous molecular mechanisms underpinning plant memory have been elucidated to date. One mechanism thereof is sustained alterations in levels of key signalling transcription factors, enzymes and/or proteins, which provides an insight into how the plant metabolism is altered and maintained by exposure to various stresses [9,10,11,12]. Another probable avenue could be chromatin alterations (DNA methylation, histone tail modifications and paused RNA polymerase II (Pol II)), which play an additional role in the coordinated changes in the patterns of gene expression that underpin memory responses [13,14,15,16]. Consequently, the underlying mechanisms of these phenomena are the subject of much research. Hitherto, the potential impact of DNA methylation induced by priming as a stress memory has been reported in numerous studies [2,17]; however, open questions remain around the specificity of epigenetic marks and their stability throughout mitosis resulting in stress memory maintenance. Moreover, the exact mechanisms linking DNA modifications to transcriptional responses under abiotic stress are still enigmatic [18]. 

Furthermore, during stress encounters, metabolic perturbations are induced as part of the defence phenomenology, and some of these metabolic responses may persist following recovery, where the plant physiology returns to equilibrium [19,20]. This change in metabolite levels such as the accumulation of signalling compounds has been shown to play a major role in stress memory under abiotic stress [21]. Thus, current models on plant stress memory point out the remodelling of molecular circuits and networks (at different cellular levels) as some of the mechanisms that define stress memory. The latter is a multi-layered complex phenomenology that implies a spatially and temporally reprogramming of the plant metabolism, involving alterations in the epigenome and metabolome [22,23]. Epistemologically, one of the most biologically best descriptions of metabolism is the metabolic profiles and fluxes it generates, representing an integrated output of the molecular and biochemical machinery of a biological system. This rises from the fact that the metabolome, being the endpoint of multi-layered biological events, carries intrinsically imprints of environmental and genetic factors [24,25,26]. Hence, the interrogation of the metabolome of a biological system—the focus of metabolomics—provides a holistic signature of the physiological state of a biological system as well as knowledge of its biochemical processes [27]. 

Furthermore, in addition to stress encounters, the plant (stress) memory or the priming phenomenology can be initiated by priming activators such as a natural or synthetic chemical compound or biostimulants. The latter are of interest for this review. The exposure of plants to these priming agents have shown to render the plant into the primed state, presenting thus opportunities for more effective use of plant priming in plant stress physiology studies and crop stress management [28]. Biostimulants are substances that, when applied in low concentrations not only mitigate stress but also promote plant growth [29]. Although the physiological effects of biostimulants have been documented [6], a comprehensive understanding of the modes of action of biostimulants at the cellular and molecular levels is still required to better value and develop formulations that are effective and science-based credible. Such in-depth molecular studies will aid the improvement of the efficacy of biostimulants and will help optimize their applications in agriculture. In the post-genomic era, comprehensive analysis using different systematic ‘omics’ approaches (systems biology) have provided insights into the complex regulatory molecular networks associated with stress adaptation and tolerance in plants. 

Thus, this review, a critical synthesis of recent literature and data, provides a current knowledge base of plant responses to abiotic stresses, highlighting key models that explain the stress perception and signalling events as well as the cellular and molecular dynamics underlying plant responses to abiotic stresses. Furthermore, attention to the epigenetic and metabolomic changes, as underlying events in plant stress memory, is reviewed, offering opportunities within the context of systems biology approaches for the alleviation of abiotic stress and induction of priming. As mentioned above, this stress memory or priming can be activated by biostimulants. In the last decade, there has been exponentially growing attention towards biostimulants as a potential solution to mitigate the negative impacts of the changing climate on agriculture, and becoming one of the pillars of a new agricultural revolution for sustainable food production. However, there is a growing need for a sound scientific foundation to pave a theoretical framework that would contribute to the formulation of biostimulant products, with scientifically-based descriptions and credibility. 

## 2. Stress Perception, Signalling and Plant Responses

To counteract the adverse effect of environmental perturbations, plants have evolved comprehensive defence mechanisms that help them tolerate abiotic stresses using physical adaptation as well as integrated molecular and cellular responses. The initial and crucial step in abiotic stress defence mechanisms is the perception of the stress signals and their transduction to activate the relevant adaptive molecular responses to ensure survival.

### 2.1. Abiotic Stress Perception and Downstream Signalling

**Figure 1:**
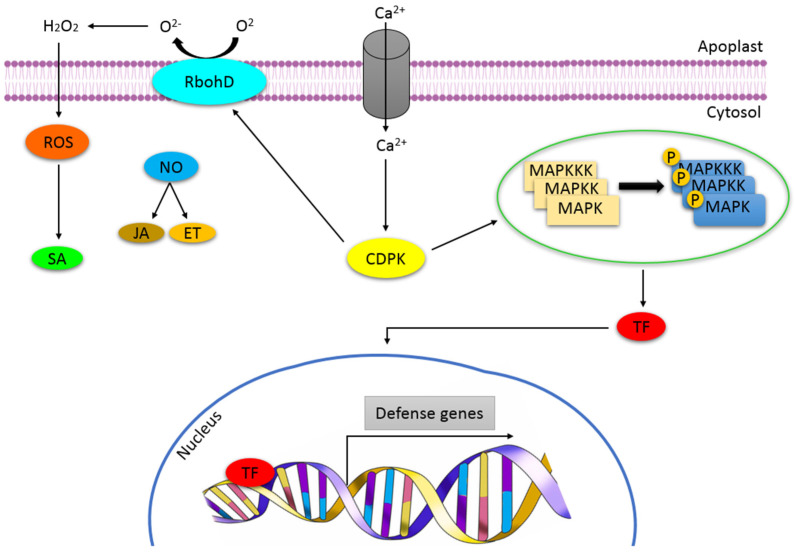
Overview of defence signalling in plants under abiotic stress. Upon perception of stress, several secondary messengers such as ROS and Ca^2+^ and NO are activated, which then induce different kinases such as CDPKs and MAPKs, resulting in the activation of transcription factors, enzymes and proteins which, in turn, activate the transcription of defence-related genes. Phytohormones, salicylic acid (SA), jasmonic acid (JA) and ethylene (ET), are also induced and contribute to plant immunity.

Perception of the stress signals is performed by receptors/sensors such as histidine kinases (HKs) and receptor-like kinases (RLKs) [30,31,32]. Numerous receptors containing leucine-rich repeats (LRR), associated with abiotic stress have been identified in plants. These receptors are classified as either receptor-like kinases or proteins (RLKs or RLPs). For the perception of abiotic stress, there is growing evidence that suggests RLKs as the main regulators of environmental stress regulation. For example, receptor-like protein kinase1 (RPK1), proline-rich-extensin-like rlk4 (PERK4), guard cell hydrogen peroxide-resistant1 (GHR1) and calcium/calmodium-regulated cysteine-rich rlk (CRK36) are involved in sensing drought and cold stress and have been reported to regulate water stress signalling in *Arabidopsis* [33,34,35,36]. Furthermore, a variety of RLKs regulates a wide range of processes including root and shoot development, symbiosis and cellular differentiation [37]. Following stress perception by the receptor proteins present on cell surfaces, the signal is transduced into different downstream signalling networks—a phenomenon known as signal transduction. A generic signal transduction pathway is initiated by perception, followed by the generation of secondary messengers resulting in the activation of a phosphorylation cascade that targets proteins involved in the regulation of stress defence genes. Early response signals have been unfolded and include cytosolic calcium (Ca^2+^) elevation [38,39], reactive oxygen species (ROS) [40,41,42] and Mitogen-activated protein kinase (MAPK) cascade activation (Figure 1).

### 2.2. Calcium Signalling in Response to Abiotic Stress

Calcium signalling plays a vital role in the specificity of the plants’ cellular responses towards stress [43,44] and each stimulus perception is followed by a rapid increase in intracellular content of the said ions. Under normal physiological conditions, resting cytosolic Ca^2+^ ([Ca^2+^]cyt) is maintained at nanomolar concentration levels via active transportation into the calcium stores or outside the cells into the apoplast, resulting in Ca^2+^ gradients of numerous magnitudes [45]. Changes in the [Ca^2+^] cyt influx and efflux patterns are evoked upon various stimuli perception as a response mechanism and this is controlled through different channels and pumps. These [Ca^2+^] cyt fluctuations by stimuli can occur in a repetitive manner in which the frequency and the amplitude of the signal are dependent on the type of the stimulus, thus making this a signature [44]. Each calcium signature encodes information that is specific to a stimulus through the type of tissue, subcellular location, size and frequency [46,47] and, therefore, defines the type of defence response. For Ca^2+^ signals to be decoded, calcium signal sensors that can sense any variations in levels and relay the information depicted within the signatures to activate relevant signalling cascades, are mandatory. Numerous studies have identified and characterised the prominent calcium sensor proteins defined by the calmodulin (CaM) and calmodulin-like protein (CMLs) family, calcineurin-B like proteins (CBLS), the calcium and calmodulin-dependent protein kinase (CCaMK) and calcium-dependent protein kinase (CDPK) family (Figure 1), which are known to occur in numerous gene families forming complex signalling networks in plants [35,48,49,50] These proteins are very diverse since they exhibit numerous affinities for Ca^2+^ ions. The binding of calcium to these sensors induces a conformational change that activates downstream targets, thereby contributing to an additional layer of specificity and resulting in the transduction of the initial stimuli perception into specific biological responses [51].

### 2.3. Reactive Oxygen Species Signalling in Response to Abiotic Stress

Abiotic stress perception triggers the production of reactive oxygen species (ROS) that serve as an early response mechanism and a crucial secondary messenger in plants. Under normal plant growth conditions, ROS are present at moderate levels through the action of antioxidants and enzymes that keep the levels in balance, thereby rendering these molecules excellent signalling transducers [52]. However, abiotic stresses induce excessive ROS production known as the oxidative burst, which may result in consequences such as cell death. ROS are defined as atmospheric oxygen intermediates that have a high biological significance in plants and include hydrogen peroxide (H_2_O_2_) (Figure 1), hydroxyl radical (·OH), singlet oxygen (^1^O_2_) and superoxide anion (O^2−^) formed through definite pathways. Accumulation of these chemical species enable plants to survive and adapt to various stress encounters [53], and even though their production differs between different cellular compartments, the generated ROS signal is still considered an abiotic stress response signature. The generation thereof is common in all stress encounters in plants and a combination of different stresses is likely to result in different ROS levels; therefore different sensors can be utilised to decode these signatures and create a signal that is specific to each stress [42,54]. Nicotinamide adenine dinucleotide phosphate (NADPH) oxidases (NOXs) catalyses the formation of superoxides and in plants are known as respiratory burst oxidase homologs (RBOHs) [55,56,57]. Phosphorylation of receptor kinases under stress encounters results in elevated Ca^2+^ which, in turn, activates the respiratory burst oxidase homolog D (RbohD) (Figure 1). Consequently, this induces excess production of ROS that causes depolarisation of plant cells walls [58]. ROS spread through the entire plant in what is known as an ‘ROS wave’ and concomitantly triggering cell-to-cell communication that results in systemic signal(s) activation [52,59]. Calcium and ROS enhance the induction of each other during stress encounters, a phenomenon known as a mutual interplay, which results in the fine-tuning of signalling [60]. For example, during salt stress, the superoxide produced activates calcium channels, which activate the vacuolar calcium channel TWO PORE CHANNEL1 (TPC1). TPC1 then transports the Ca^2+^ from the vacuole and induces the activation of RBOH protein D. This feedback loop is responsible for the propagation of the ROS and Ca^2+^ waves resulting in an efficient acclimation response [61].

### 2.4. Mitogen-Activated Protein Kinase Pathway Activation in Response to Abiotic Stress

In addition to rapid systemic signalling induced by Ca^2+^ and ROS secondary messengers, kinase cascades of the MAPKs similarly play a crucial role in plant signalling of several environmental cues. The MAPK cascade is a result of a series of phosphorylation events that activate relevant genes in response to different stresses. In this system, the induced stress signals are transported from the receptors to specific effectors, thereby resulting in the regulation of relevant genes, different cellular activities and proteins involved in development and adaptation processes [62,63,64,65]. Signal transduction by MAPK cascades encompasses three types of kinases namely MAPK, mitogen-activated kinase kinase (MAPKK) and mitogen-activated kinase kinase kinase (MAPKKK) (Figure 1). Firstly, the MAPKKK, located downstream of specific receptors, is activated in response to extracellular stimuli which, in turn, activates a downstream MAPKK via phosphorylation of its two serine or threonine residues located in the activation loop. Subsequently, MAPKK behaves as a specific kinase and phosphorylates a MAPK on its tyrosine or threonine residues located in the activation loop [64,66]. The latter eventually leads to the activation of various effector proteins located in the nucleus or cytoplasm as well as additional protein kinases, enzymes or transcription factors involved in plant stress-driven signalling pathways (Figure 1). Furthermore, MAPK cascades activated upon abiotic stresses such as drought, cold and salt show cross-talks with secondary messengers (ROS, Ca^2+^ and nitric oxide (NO)) and phytohormones [67,68]. The integration of these signalling events mediated by the interplay (synergistically or antagonistically) between ROS, phytohormones and other signalling molecules choreographs abiotic stress responses and drives the changes in the transcriptomic, metabolomic and proteomic networks that lead to acclimation and survival [69]. 

Cross-talks are defined as converging points among signal transduction cascades that work collaboratively to convey and integrate stress stimuli and ultimately orchestrate processes such as plant growth, development and abiotic stress defence responses [68,69]. Phytohormones are amongst the most crucial signalling molecules that regulate these growth and development processes [70,71]. Phytohormones interact by activating secondary messengers and phosphorylation cascades executed by MAPKs. These transduction cascades result in the regulation of gene expression that directly influences the biosynthesis of different phytohormones. Perception, signal transduction and phytohormone synthesis, therefore, creates complex crosstalk in which different signals are integrated and numerous MAPK cascades respond to phytohormones such as abscisic acid (ABA), jasmonic acid (JA), salicylic acid (SA), ET and auxins. Usually, each signalling molecule participates in distinct signalling pathways, resulting in the formation of a cross-talking network that coordinates responses to different abiotic stresses [72]. 

### 2.5. Phytohormone Production in Response to Abiotic Stress

During stress exposure, plants can amplify the initial stress signals, depending on the type of stress encountered and they do this by making use of phytohormones. These phytohormone-driven signalling can either trigger new signalling events that are similar to those of the initial signal or initiate an entirely different signalling event with different components [73]. The most reported plant defence response phytohormone against abiotic stresses is ABA. Phytohormone accumulation has been linked to early plant stress signalling events such as rapid ROS production, showing the importance of the early and conventional plant responses in phytohormonal regulation that is dependent on the nature of the stress. For instance, the accumulation of ABA under water deficit and high salinity is dependent on ROS production via the NADPH oxidase [74]. This ABA-induced ROS accumulation can enter guard cells and activate Ca^2+^ channels which result in an increase in [Ca^2+^]cyt and thus induce stomatal closure [75]. ABA is first sensed by the cells via the ABA receptors RCAR/PYR1/PYL (Regulatory Components of ABA-receptor/pyrabactin resistant protein/PYR-like proteins) [76], resulting in the activation of open stomata 1 (OST1). OST1 is a member of the SNF1-related protein kinase 2 (SnRK2) family that mediates ABA-induced stomatal closing and the regulation of ROS production through the phosphorylation of the NADPH oxidase [77]. MAPK cascades have also been implicated in ABA-mediated stress responses that are either upstream or downstream of ROS production [78]. These signalling events lead to the activation or initiation of various cellular/molecular defence mechanisms characterized by defence genes, proteins and metabolites. 

### 2.6. Abiotic Stress Responses: Cellular and Molecular Events

Plant defence responses, aiding in adaptation to abiotic stresses, are coordinated by networks of cellular and molecular mechanisms, fine-tuning changes in the growth and development of the plant. Significant progress has been made in elucidating these plant defences against environmental perturbations, which generally comprise alterations in the plant transcriptome, proteome and metabolome [79,80,81,82]. Following signal perception and transduction, adaptive responses are activated and result in the expression of stress-related genes regulated by transcription factors (TFs) at the transcriptional level (Figure 1). A single TF can regulate the expression of numerous genes through the specific binding thereof to the *cis*- and *trans*-element in the promoters of target genes and this type of transcriptional regulation is termed regulon [83]. In this instance, several genes are regulated in response to the same signal and regulatory protein and any environmental stimulus can induce several regulons. 

**Figure 2:**
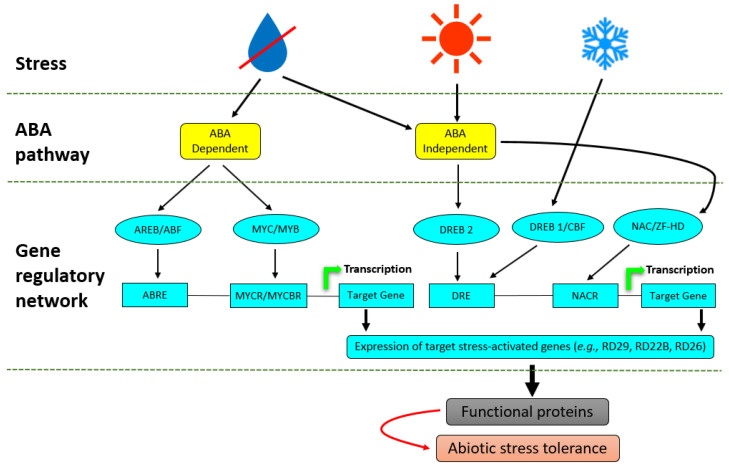
Transcriptional regulatory networks of abiotic stress signals. Signal transduction pathways in drought, heat and cold-stress responses are either ABA-dependent or ABA independent. In the ABA-dependent pathway, ABRE functions as a main ABA-responsive element. MYB2 and MYC2 function in ABA-inducible gene expression of the *RD22* gene. MYC2 also functions in JA-inducible gene expression. The RD26 NAC transcription factor is involved in ABA and JA-responsive gene expression in stress responses. DRE is mainly involved in the regulation of genes not only by drought and salt but also by cold stress. DREB1/CBFs are involved in cold-responsive gene expression. DREB2s are important transcription factors in dehydration and high salinity stress-responsive gene expression. Another ABA-independent pathway is controlled by drought and salt, but not by cold.

Numerous regulons that are activated in response to abiotic stress have been identified in plants and are components of ABA, a principal phytohormone involved in the regulation of abiotic stress in plants by regulating an intricate gene regulatory system that permits plants to tolerate environmental perturbations [84]. Myeloblastosis oncogene (MYB)/myelocytomatosis oncogene (MYC) and the ABA-responsive element-binding protein/ABA-binding factor (AREB/ABF) regulons function in ABA-dependent gene activation pathways [85], whereas dehydration-responsive element-binding protein 1 (DREB1)/C-repeat binding factor (CBF), DREB2, NAC (CUC, NAM and ATAF) and the zinc-finger homeodomain (HD) regulons function in ABA-independent gene expression (Figure 2). The different TFs involved in stress tolerance normally function independently of each other; however, it has been shown that the ABA-dependent and ABA-independent pathways converge at several points representing transcriptional repressors and enhancers which may interact directly or indirectly with the DREB and AREB, and hence initiate synergistic interactions between cold, drought and salinity stress [86,87].

Abiotic stress-inducible genes that are regulated by the different regulons include late embryogenesis abundant (LEA) class genes (*RD29B*, *RAB18*), cell cycle regulator genes (*ICK1*) and PP2Cs (*ABI1* and *ABI2*) (Figure 2). RD22 and RD26 have received special attention as potential targets for the improvement of abiotic stress tolerance [88]. These stress-regulated genes together with their products have important roles in abiotic stress responses and tolerance such as their translation into functional proteins which regulate numerous abiotic stress defence responses such as osmolyte accumulation, membrane protection, ROS-scavenging and stomatal closure. Much progress has been made in the understanding of signal transduction, transcriptional regulation and gene expression in plant responses to different abiotic stresses [89,90]. In transgenic *Arabidopsis thaliana* for example, the overexpression of Glycine soja NAC TF, designated as GsNAC019, induced alkaline stress tolerance at both the seedling and mature stages, even though the transgenic plants had reduced sensitivity to ABA [91]. In addition to all the plant defence responses induced by abiotic stress previously mentioned, plants can be primed for more rapid and stronger defence responses towards stress, and the emergence of microbial biostimulants as potential priming agents has been exponentially grown. 

## 3. Microbial Biostimulants and Enhancement of Plant Responses to Abiotic Stresses

Plant growth and development regulation, together with the alleviation of detrimental effects of abiotic stresses, are crucial factors that determine the productivity of cultivated plants. Abiotic stresses are well known to negatively affect plant growth and development and are responsible for crop losses globally. However, the current knowledge on molecular and cellular mechanisms involved in mitigating these deleterious effects are still limited. Furthermore, biostimulants are increasingly being integrated into production systems, to modify underlying plant physiological and biochemical processes to enhance stress tolerance and productivity. By definition, biostimulants are diverse substances or microorganisms that are formulated to stimulate the plant’s natural processes to enhance plant productivity through different modes of action, irrespective of their nutrient content or (individual) constituents [29,92]. Additionally, biostimulants foster plant growth and development throughout the plant’s life cycle from seed germination until maturity, improve the plant’s metabolism, improve stress tolerance, facilitate nutrient assimilation, translocation and utilisation, enhance soil physiochemical properties and drive the development of complementary soil microorganisms [93].

Plant biostimulants are available in a wide range of formulations with varying constituents but are generally categorised into four major groups based on their source and content. These groups include amino acid-containing products (AACP), hormone-containing products (HCP), humic substances (HS) and plant growth-containing microorganisms [29]. Additionally, numerous categories of biostimulants have been extensively reviewed including protein hydrolysates [94], seaweed extracts (SWE) [95], silicon [96], humic and fulvic acids [97], arbuscular mycorrhizal fungi [98] and plant growth-promoting rhizobacteria (PGPR) [99]. In this regard, the potential effects of some of these biostimulants in ameliorating abiotic stress in various plants have been extensively reviewed (Table 1).

Several possible key mechanisms of action induced by biostimulants in relation to abiotic stress alleviation have been elucidated and include ROS scavenging, membrane stability, osmoprotection, stomatal regulation, ion homeostasis, nutrient availability and metal chelation [120], however, the explicit underlying modes of action responsible for these effects remain largely unknown [92,121].

### 3.1. PGPR-Based Biostimulants and Defence Priming Against Abiotic Stresses

PGPR are increasingly being used as biostimulant formulations, showing potentials for improving plant health, development and sustainable increased yield. These soil bacteria that inhabit the rhizosphere interact symbiotically with the plant host to enhance plant growth. This chemical communication is translated into physiological benefits through various mechanisms which include nitrogen fixation, production of growth-stimulating phytohormones and solubilisation of mineral phosphates [122]. A detailed account of the complexity of the rhizosphere, its densely and diverse population and molecular signalling web [123,124,125,126] is beyond the scope of the current review. Although the rhizosphere chemistry remains largely unknown and the establishment of plant-rhizomicrobiome mutualistic interactions is still poorly characterised, emerging studies have reported that various PGPR species enhance improvement in agronomic yields through direct or indirect mechanisms [127,128,129], which include nitrogen fixation, production of growth-stimulating phytohormones and solubilisation of mineral phosphates [122]. Similarly, according to numerous studies (Table 2), the improvement in agronomic yields by PGPR (or PGPR-based biostimulants) is due to the production of growth-stimulating phytohormones such as indole-3-acetic acid (IAA), zeatin, ABA, ET and gibberellic acid (GA), secondary metabolites (siderophores, lipopeptides and N-acyl homoserine lactone) and volatile organic compounds (hydrogen cyanide, acetoin and 2,3 butanediol). Most recently, it has been demonstrated that *Azospirillum brasilense* Sp245 lipopolysaccharides (LPS) stimulate growth (fresh weight and root length) in *Arabidopsis thaliana* [130] and this further suggest that PGPR-derived MAMPs also play a role in plant growth stimulation.

**Figure 3:**
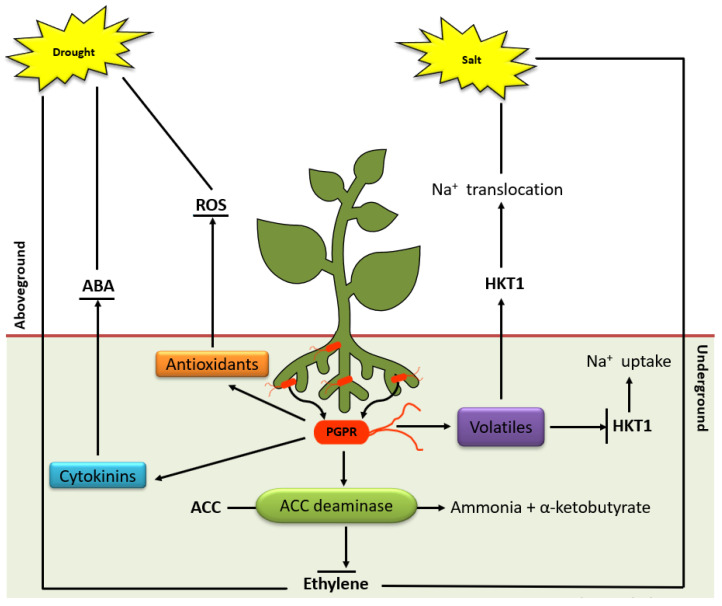
Overview of induced systemic tolerance elicited by PGPR against drought and salinity **stress**. Broken arrows indicate compounds secreted by PGPR under stress including cytokinin, antioxidants, ACC deaminase and volatiles. Cytokinins and antioxidants result in ABA accumulation and ROS degradation, respectively. ACC deaminase degrades ACC and inhibits ET production. The volatiles emitted by PGPR downregulates HKT1 expression in roots but upregulation in shoot tissue, resulting in the recirculation of Na+ in the whole plant under high salt conditions.

The application of PGPR to induce abiotic stress tolerance in plants is increasingly being explored as an attractive strategy to regulate plant stress [142,143,144] and several mechanisms through which these organisms induce stress tolerance have been deciphered (Figure 3). In addition to the plant growth mechanisms mentioned above, PGPR can also induce abiotic stress tolerance in plants by modifying phytohormonal activity, maintaining iron homeostasis and osmotic balance [138,139].

Under abiotic stresses PGPR employs different mechanisms to help plants survive the environmental changes including (i) production of ACC deaminase which lowers ET levels in plants [145,146]; (ii) osmolytes secretion (proline, choline and trehalose) which acts as osmoprotectants; (iii) bacterial volatile secretion to induce stress tolerance (i.e., 2R,3R-butanediol induces stomatal closure); (iv) secretion of phytohormones (IAA, gibberellins and cytokinins) which stimulates lateral roots and root hairs formation, thus, increasing water and nutrient uptake; (v) changes root cell membrane elasticity and improve membrane stability; (vi) exopolysaccharide secretion, which improves permeability by increasing soil aggression and maintaining high water potential around plant roots. Besides these known mechanisms, PGPR can trigger physiological events/processes regulated by a complex network of signalling events consequently ensuing stress tolerance and priming [5,147,148].

### 3.2. PGPR-Induced Priming as Strategy towards Enhanced Abiotic Stress Tolerance

**Figure 4:**
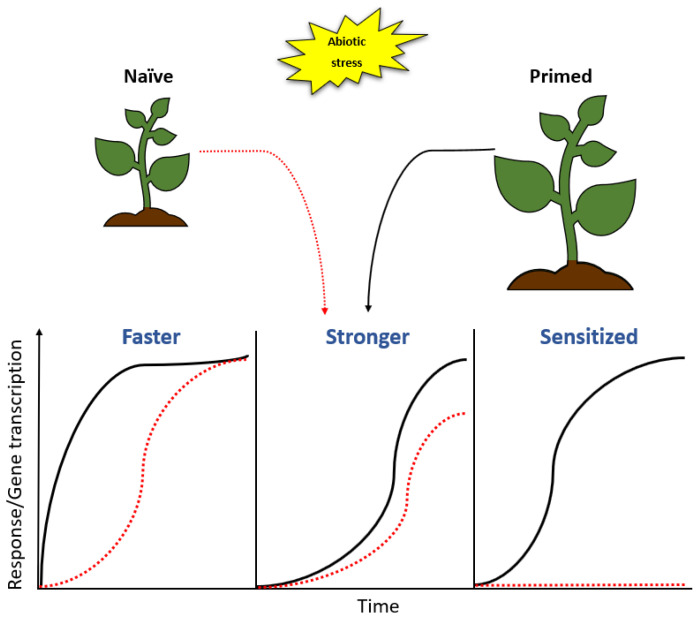
Priming modifies responses upon stress encounter. A naïve plant may be primed by either exposure to stress or other priming factors such as microbes. Response patterns differ in primed and naïve plants; the primed plant may respond to inducing stress more rapidly or more robustly than a naïve plant. It may also be sensitised so that the response is triggered at a lower fitness cost. The primed plant may further modify its response mechanisms to regulate a network of genes that is different from that found in a naïve plant.

PGPR-plant interactions lead to enhanced resistance against abiotic stresses via PGPR-induced preconditioning of the plant immunity and defences, a phenomenon known as priming. In this state, the plant responds more rapidly and/or robustly following exposure to stress, thereby resulting in better stress tolerance when compared to non-primed plants (Figure 4). This condition of preparedness achieved termed the ‘primed state’ has been linked to efficient activation of the defence responses which result in enhanced stress resistance. The enhanced resistance can be characterized by various mechanisms such as systemic acquired resistance (SAR) and induced systemic resistance (ISR). SAR is a defence response mechanism activated in distal parts of the plant upon localized infection [149] and it confers resistance to subsequent stress encounters, thus priming the plant to defend itself upon attack. ISR on the other hand is mediated by micro-organisms that mediate plant growth such as PGPR, which induce resistance in the plant by colonizing the root system [150,151]. SAR induction is SA dependent [152], whereas ISR employs ET and JA response pathways to induce resistance. The emergence of SAR and ISR as crucial modes of priming has been widely reported [153,154,155,156], however, a grey area still exists through which these priming mechanisms take place. Even though priming mechanisms are not fully understood, numerous hypotheses have been proposed and include the accumulation of inactive proteins involved in signal amplification such as MAPKs [157], activation of transcription factors that enhance transcription of defence-related genes following stress perception [4] and epigenetic changes involving DNA modifications, histone modifications or chromatin alterations [158]. Plant priming has been considered as a promising strategy for the control of stress because it enhances defence responses without affecting the overall fitness of a plant and the resultant stress resistance or tolerance cannot be overcome by microbes, subsequently providing long-term resistance [159].

The rhizosphere chemistry and the development of plant-rhizomicrobiome interactions are still poorly characterised, however, emerging studies have reported that various PGPR species can pre-condition the plants for augmented defence responses against abiotic stresses [160,161,162,163,164]. The molecular mechanisms underlying the rhizobacteria-related defence priming show that this induced state suggests a reprogramming in the cellular metabolism and regulatory machinery of the plants. Current insights propose that, preceding environmental perturbations, primed plants re-programme metabolic pathways by altering the biosynthesis of various compounds such as sugars, amino acids and tricarboxylic acids [3,165]. Recent studies have reported on the ability of PGRP-induced priming to enhance responses to abiotic stresses. For example, Carlson et al. [166] showed that PGRP treatment-induced differential metabolic reprogramming correlating to enhanced drought stress tolerance in *Sorghum bicolor*. The underlying metabolic changes in the enhanced drought stress tolerance induced by PGPR-primed *S. bicolor* plants included augmentation of the antioxidant system, root architecture modifications, activation of induced systemic tolerance and production of the osmolytes. Under salt stress, Singh and Ja [167] reported on the ability of PGPR in enhancing defence responses resulting in stress tolerance. Wheat plants inoculated with PGPR showed a significant increase in plant growth (shoot length/root length, fresh weight/dry weight and chlorophyll content. Additionally, PGPR inoculation elevated the antioxidative enzyme activities of superoxide dismutase, catalase and peroxidase. A significant decrease in the accumulation of Na^+^ in both shoots and roots and an increase of K^+^ uptake was observed, thereby favouring the K^+^/Na^+^ ratio which mitigates salinity induced oxidative stress. 

Even though progress has been made on the elucidation of PGPR-induced priming, the knowledge of biochemical and molecular mechanisms in defence priming is still largely unknown and a detailed mechanistic description of the various layers driving the priming events is still limited [168]. Nevertheless, despite these limitations, this potentiation of the immune system and stress adaptability is unquestionably a fundamental means underlying plant defence responses that are amplified, resulting in increased stress tolerance. As such, defence priming represents a promising and complementary alternative strategy that can provide new opportunities for plant protection against abiotic stress. Priming mechanisms are described at different levels and can either be long- or short-lived. DNA methylation and histone modification observed at the epigenetic level are long-lasting mechanisms [169] and modulation of enzyme activities and changes in the abundance of transcripts observed at the transcript or protein level are short-lived mechanisms [22]. The process of priming reconfigures the long-lasting mechanisms resulting in the plant genome retaining an “epigenetic memory” which is induced upon abiotic stress challenges allowing for a rapid expression of the primed responses. 

## 4. Plant Epigenetic Mechanisms and Their Role in Abiotic Stress Responses

**Figure 5:**
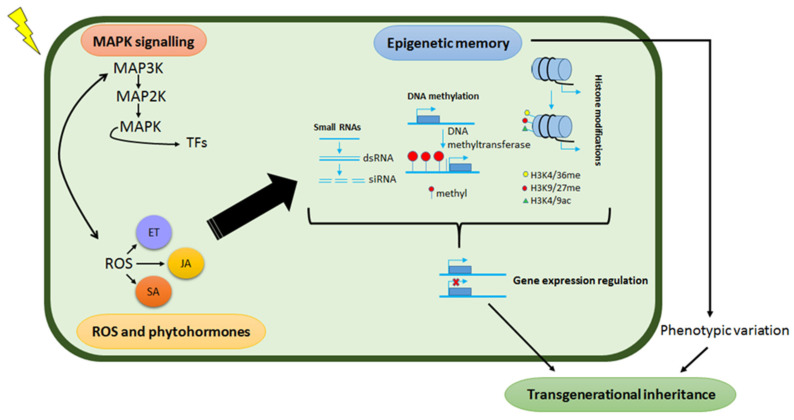
Simplified model of abiotic stress signalling pathways and their role in transgenerational epigenetic memory. Perception of environmental stress by plants induces the accumulation of ROS, which in turn promotes the production of phytohormones including SA, JA and ET. ROS further induce a MAPK signalling cascade that activates transcription factors, driving defence-related gene transcription. These stress-induced changes modulate the epigenetic landscape (small RNAs, DNA methylation and histone modifications) which subsequently affect gene expression patterns in response to abiotic stress. These epigenetic variations can be transient and revert to the initial epigenetic state. In some cases, induced epigenetic variations may be transmitted transgenerationally and may become adaptive if the offspring experiences environmental signals similar to the previous generation.

Epigenetics refers to heritable changes in gene expression without alterations in the underlying DNA sequence and a growing number of studies postulate such regulations to be part of the underlying mechanisms of priming effects [21,170,171]. This epigenetic remodelling includes histone modification as well as small RNAs and DNA methylation events which participate in the regulation of stress-responsive genes at both the transcriptional and post-transcriptional levels by altering the chromatin status of the genes. Moreover, epigenetic modifications play crucial roles in the formation of stress memory, which may be inherited by the progeny resulting in enhanced stress tolerance. Some of these modifications persist longer and are considered as transgenerational ‘memory marks’, whereas others are short-lived, dynamic modifications that are quickly removed again—i.e., chromatin marks [10,22,172,173,174]. Although the molecular workings that define the transgenerational primed state are still largely unknown, epigenetic modifications and imprints are key components of this cellular and molecular phenomenology of stress memory for storing and retrieving stress-related information [17]. As such, epigenetic transgenerational memory in plants is defined as a memory mark that extends from a generation under stress exposure to the first generation not exposed to the same stress (Figure 5).

Pecinka and Mittelsten Scheid [10] cogently argued that the evidence of transgenerational epigenetic inheritance resultant from abiotic stress requires long-lasting changes in two generations or more that significantly influenced the plant’s stress tolerance. Conversely, Yaish [175] suggested that there is evidence for long-lasting epigenetically-induced changes in stressed plants, however, it is difficult to spot. Consequently, for the past few years, several studies have reported on how plants acquire new traits induced by stress cues due to changes in epigenetic marks observed in the transposable elements (TEs) [176], promoters [177] and gene coding regions [178]. It has been proposed that transgenerational inheritance of epigenetic marks and stress tolerance form part of the adaptive process in plants [11] through the transfer of epigenetic modifications from the parental genome to the progeny without any reprogramming occurring in the gametes and embryos. The degree of this reprogramming is, however, still blurred, thus leaving a question of how much of the stress-induced epigenetic marks induced by priming are transferred to the progeny. Key common molecular features underlying epigenetic modifications associated with priming in abiotic stresses have been reported in numerous studies and include transcriptional memory, histone methylation and DNA hypo- and/or hypermethylation [179,180,181,182] (Table 3).

Zheng et al. [198] reported the improvement of drought adaptability in rice plants due to multi-generational drought exposure. They identified drought-induced epimutations, which could maintain altered DNA methylation levels in the subsequent generations. Analysis of the drought-associated genes revealed that the DNA methylation level of the genes was modified by the multigenerational drought stress. These results, therefore, suggest that epigenetic mechanisms play imperative roles in plant’s adaptations to environmental stresses. Consequently, the heritable epigenetic variations having morphological, physiological and ecological consequences can be considered important resources in plant improvement which may help improving adaptation and tolerance in crop plants for the adverse environments.

### 4.1. DNA Methylation and Plant Responses to Abiotic Stresses

DNA methylation is an epigenetic mark that involves the transfer of a methyl group from S-adenosyl methionine (SAM) to the fifth carbon of the pyrimidine ring of cytosine nucleotide without altering the underlying DNA sequence [199]. The modification machinery involves a highly regulated series of enzymatic reactions and complex molecular rearrangement events. DNA methylation is generally catalysed by a variety of enzymes termed DNA methylases (MTases) (Figure 5), comprising two main groups: (i) de novo MTases, which catalyse the transfer of a methyl group to unmethylated cytosines, and include methyltransferase 1 (MET1), chromomethylase 3 (CMT3) and domains rearranged methyltransferase (DRM); and (ii) maintenance MTases, which maintain methylation that has already been established [200]. This epigenetic mechanism is involved in various biological processes such as development, stress adaptation and genome stability and evolution. Methylation of cytosine bases can be symmetric CG and CHG, and asymmetric CHH (where H = A, C or T) [190,201,202]. Symmetrical methylation involves the recruitment of a MET to hemimethylated daughter strands following DNA replication. Conversely, asymmetric methylation is determined de novo after every replication cycle and does not possess any inheritance mechanisms [17]. In plants, de novo methylation is catalysed by DRM2, and maintained by three different pathways: CG methylation by MET1, CHG methylation by CMT3, a plant-specific DNA methyltransferase and asymmetric CHH methylation through persistent de novo methylation by DRM2 [203]. Studies have revealed that in plants, methylation occurs predominantly on the CG, then CHG and CHH context, respectively [204]. In addition, DNA methylation in plants is usually restricted to CGs located within the gene body while TE sequences tend to be methylated at most of their CG, CHG and CHH sites [205,206]. The highly abundant methylation on repetitive DNA sequences suggests that one of the main functions of this epigenetic mechanism is to suppress the activity of transposons.

TEs make up a considerable proportion of the plant’s genome, therefore the regulation thereof is essential because they are potentially highly mutagenic and their accumulation limits survival [207]. Moreover, DNA methylation is also observed in gene coding regions in plants and often assembled in regulatory regions of genes such as promoters where it plays a vital role in regulating gene expression. Numerous studies have reported that methylation in the promoter region causes transcriptional silencing, suggesting that changes in methylation could lead to novel transcriptional regulation of the associated genes [208,209]. As such, changes in DNA methylation contribute tremendously to the plant’s capability to conquer and respond to adverse conditions [210]. Under stress conditions, changes in hypermethylation of DNA (an increase in epigenetic DNA methylation) or hypomethylation of DNA (a decrease in methylation of DNA) [211] result in gene expression alterations (activation/suppression) and are indicative of stress defence mechanisms, however, this is dependent on the type of stress response induced and varies between species. Given the changes orchestrated by DNA methylation, which result in gene silencing/activation, plants possess enzymes that counteract the activity of MTases to remove the methylation—a process known as DNA demethylation. DNA demethylases demethylate TEs or transcription start sites for gene expression regulation. These enzymes include demeter (DME), repressor of silencing 1 (ROS1) and demeter-like (DML) proteins (DML2 and DML3), which initiate demethylation via a base excision repair (BER) mechanism [212,213,214]. 

Several examples have indicated that DNA methylation can either increase or decrease in response to stress and it appears that demethylation, which leads to gene expression activation, is a prompt response common in plants. In maize seedlings under cold stress exposure, hypomethylation was observed in root tissue following the expression of ZmMI1 and after seven days of cold exposure (recovery), the cold-induced decrease in methylation levels was not restored to basal levels [193]. Additionally, in *Populus trichocarpa*, an increase in methylation was observed under drought stress, with 10.04% compared to the watered plants with a 7.75% increase [194]. Herein, it has been reported that there is a correlation between stress-induced gene expression and methylation levels under drought stress, with an up-regulation of 7329 genes together with hypermethylation as well as 10 322 down-regulated genes with hypomethylation shown [194]. Methylation status/level differs between species and genotypes under different stress factors. For example, an increase in DNA methylation levels was reported in different rice genotypes of 20% in Nagina-22 and 37% in IR-64-DYT1.1 under drought stress [215]. The accumulated knowledge on DNA methylation over the years has been a big accomplishment in plant biology and to understand this phenomenon better, the question to ask is whether adaptation and stress memory are determined by particular site-specific methylation or methylated regions and on how these regions are regulated by defence signalling. 

Furthermore, emerging studies have evidently pointed to DNA methylation as one of the key components of stress priming and memory [2,17,182,216,217]. Recently, Sun et al. [218] demonstrated that drought induces rapid desiccation tolerance (RDT) that can be maintained for at least four weeks. Comparison of genome-wide DNA methylation levels revealed a dehydration stress-responsive hypomethylation in the CG, CHG and CHH contexts and acclimation-induced hypermethylation in the CHH context of the *Boea hygrometrica* genome which was consistent with the transcriptional changes in methylation pathway genes. As expected, the global promoter and gene body methylation levels were negatively correlated with the gene expression levels in both acclimated and dehydrated plants. Nonetheless, the promoter methylation variations in the CG and CHG contexts were significantly associated with the differential expression of genes required for fundamental genetic processes of DNA conformation, RNA splicing, translation and post-translational protein modification during acclimation, growth and rapid dehydration stress response. Additionally, the observed DNA methylation changes were associated with the upregulation of stress memory genes including pre-mRNA-splicing factor 38A, vacuolar amino acid transporter 1-like and UDP-sugar pyrophosphorylase, which may contribute to the enhancement of dehydration tolerance in *B. hygrometrica* plants. 

### 4.2. Histone Modifications, Small RNAs and Abiotic Stress Responses

In addition to alterations of DNA molecules through methylation or other processes, the chemical modification of histones is another main component of the epigenome. Histones are essential proteins involved in the packing and ordering of the DNA molecule into a fundamental structural unit of chromatin called a nucleosome. The latter consists of ~147 bp of DNA wrapped around a histone octamer that contains two copies each of H2A, H2B, H3 and H4, and accessibility of genomic DNA is regulated at this core particle [219]. The N-terminal regions (or tails) of the histones protrude from the larger structure and are prone to various reversible, chemical post-transcriptional modifications (PTMs) that affect chromatin structure and function [14]. Histone PTMs involved in enhancing/repressing gene expression include acetylation, phosphorylation, ubiquitination, biotinylation, de-acetylation, sumoylation, carbonylation and glycosylation catalysed by various enzymes. These histone modifications thus alter gene accessibility for transcriptional machinery [220,221,222,223,224]. 

In the context of plant-environment interactions, studies have revealed that histone modifications that are closely related to plant responses to abiotic stress exposure are histone acetylation, de-acetylation, methylation and demethylation, which are catalysed by histone acetyltransferases (HATs), histone deacetylases (HDACs), histone methyltransferases (HMTs) and histone demethylases (HDMs), respectively [225,226]. The trimethylation of H3K4 is reported to be associated with transcription activation whereas dimethylation of H3K9 and H3K27 represses gene transcription [227]. For instance, in *Arabidopsis thaliana*, a reference plant generally used for epigenetic studies in plant responses to abiotic stresses, under prolonged cold stress, an increase in H3K9 and H3K27 dimethylation and a decrease in H3K4 trimethylation was observed [228]. Furthermore, histone acetylation takes place at the flowering locus C (FLC), a flowering repressor. These histone modifications result in stable repression of FLC that permits flowering in winter-annuals types of *A. thaliana* that exhibit flowering delays in the first season [228]. Sokol et al. [229], using western blotting to study the response of Arabidopsis T87 and tobacco BY-2 cell line nucleosomes, demonstrated that phosphorylation and phosphoacetylation of histone H3 Ser-10, as well as acetylation of histone H4 lys14, were increased under cold stress and high salinity conditions. 

Similarly, to DNA methylation, histone modifications as probable marks of priming and stress memory have been reported in several studies [170,181,230,231,232]. A recent study by Liu et al. [233] reported that extreme heat-stressed Arabidopsis plants showed accelerated flowering which was also observed in the unstressed offspring, however, the mechanism remains unknown [234]. They further showed that the heat-induced heat shock transcription factor (HSFA2) activated the H3K27me3 demethylase relative to early flowering 6 (REF6) which de-repressed HSFA2. The REF6 and HSFA2 form a loop that activates transgenerational degradation by the suppressor of gene silencing 3 (SGS3)-interacting protein 1 (SGIP1) which, in turn, leads to the biosynthesis of trans-acting siRNA (tasiRNA) inhibition. The REF6-HSFA2 loop induces early flowering but decreases immunity. This feedback loop is a form of long-term epigenetic memory of heat that is maintained by the transgenerational ‘ON’ state of HSFA2. In addition to histone modifications, the stalling of RNA polymerase II was suggested as a drought stress-induced memory mark in *A. thaliana* [179] and could provide a chromatin content that is active and prepares genes involved in development and stimuli responses for appropriate expression. 

Target gene repressions by small non-coding RNAs (Figure 5) have also been found to be engaged during plant abiotic stress and transcriptional gene silencing (TGS) by 24 nucleotides heterochromatic small interfering RNAs (hc-siRNAs), via RNA directed DNA methylation (RdDM), which is reported as an epigenetic mechanism of gene regulation in plants [202,235]. RdDM in plants is exclusive to small RNA-mediated chromatin modifications because it depends on particular transcriptional machinery that is fixated around two plant-specific RNA polymerase II (Pol II)- related enzymes called Pol IV and Pol V [236]. In brief, the canonical view of RdDM involves the following steps: (i) transcripts from Pol IV are first copied into long double-stranded RNAs (dsRNAs), (ii) processed by dicer-like 3 (DCL3) into siRNAs and transported to the cytoplasm, (iii) following loading of one strand of these siRNAs onto Argonaute (AGO4), they are re-imported to the nucleus, where the siRNA guides the targeting of transcripts from Pol V by sequence complementarity and (iv) ultimately, this targeting recruits DNA methyltransferase activity to mediate de novo methylation of cytosines in all classes of sequence contexts [202]. Numerous examples of environmentally responsive siRNAs as key mechanisms for priming and stress have been reported [176,237,238], however, the link and correlation between epigenetic modifications and acclimation is still an ongoing challenge. 

Evidence on epigenetic modifications induced by microbial biostimulants has been reported by Gagné-Bourque et al. [239] in which plant-growth-promoting bacteria (PGB) Bacillus subtilis B26 induced an increase of 6-fold and 1.5-fold of global DNA methylation in model grass *Brachypodium distachyon* under normal plant growth conditions. The reported hypermethylation was correlated to an increase in the abundance of methyltransferases involved in the maintenance and regulation of DNA methylation. Additionally, the PGB induced hypermethylation levels remained constant when compared to the naïve plants after five and eight days of drought treatment, suggesting that *Bacillus subtilis* B26 potentially acts at an epigenetic level to increase drought stress tolerance in *Brachypodium distachyon* by inducing DNA methylation changes under normal conditions (priming) that, in turn, increase drought resistance by allowing the expression of drought-responsive genes. De Palma et al. [240] very recently reported on the ability of *Trichoderma harzianum* (a fungus used in biostimulant formulations) to induce epigenetic modifications, namely DNA methylation, providing an insight into plant-microbial interplay in tomato roots. The results showed a decrease in DNA methylation levels at the different time points, which correlated to transcriptional regulation of defence-related genes. The observed epiregulator expression mediates plant defence mechanisms by the interaction with *Trichoderma* and/or other *Trichoderma*-induced effects. Recent studies have extended our understanding of epigenetic modifications induced by microbial biostimulants; however, the majority of these studies mainly focused on epigenetic mechanisms associated with microbial biostimulants under biotic stress [241,242,243,244] and thus, the investigation of abiotic stress-induced epigenetic mechanisms is still largely unexplored. In addition to epigenetic modifications that result from the complex multi-layered defence responses induced by abiotic stress, primary and secondary metabolites are also altered as a form of metabolomic adaptation. As such, the crosstalk between epigenetics and metabolism are fundamental aspects of cellular adaptation to abiotic stress. The epigenome is dynamically regulated by the metabolome and alterations in either arising from abiotic stress cues may therefore coordinately drive aberrant gene expression which, in turn, contributes to adaptation and stress memory (epigenetic and metabolomic memory). 

## 5. Metabolomics and the Elucidation of Plant Responses to Abiotic Stresses

Various cellular and biochemical changes induced by abiotic stress are defined at the plant metabolism level. In a simplified description, under abiotic stresses, the plant metabolism is perturbed due to factors such as inhibition of enzymes or increased demand for compounds required for normal growth and development. Consequently, the plant’s metabolic network must continually readjust under these unfavourable conditions to maintain the normal metabolomic homeostasis and allow for the production of defence-related compounds that aid in stress tolerance. Accordingly, the current implementation of metabolomic approaches provides a thorough analysis of vital components of the plant’s defence responses to abiotic stress. Here, metabolomics is a powerful tool that provides an overview of how an organism’s metabolic network is regulated in response to stimuli. Metabolomics is a rapidly expanding *omics* science that has been widely applied in different fields. This multidisciplinary *omics* science is defined as the comprehensive, qualitative and quantitative analysis of all the small molecules, termed metabolites, in a biological system [245,246]. 

**Figure 6:**
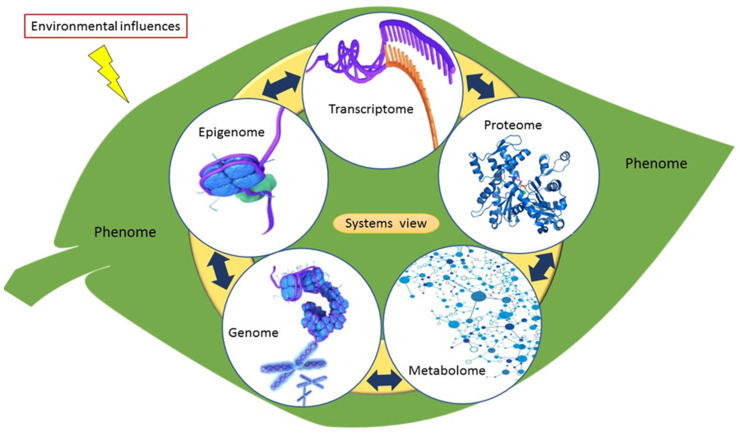
Systems overview and interconnectedness of cardinal *omics* approaches. The metabolome is the downstream output representing the physiological state of the phenotype resulting from the direct flow of biological information. The downstream output represented by the metabolome not only reflects the integration of the genome, epigenome, transcriptome and proteome output, but also the input from environmental influences such as abiotic stress.

The metabolome comprises a pool of low-molecular-weight metabolites usually less than 1500 Da. These metabolites are considered final products of cellular regulatory processes (gene expression and protein activity), modulating processes between the genome and the environment. In this regard, metabolomics is a cornerstone in the integration of the ‘-omics’ technologies that contribute to a systems biology overview (Figure 6). As such, it assists in providing a holistic understanding of the organisation principle of cellular functions at different biological information levels and in providing ways of monitoring biological processes in an integrated system [27] (Figure 6). Metabolites form an indispensable part of the plant metabolism, influencing all biological processes such as plant defence or tolerance to abiotic stresses [247]. As a high-throughput technology, metabolomics has been extensively used for various studies ranging from drug discovery, enzyme discovery, nutrigenomics, microbial biotechnology, toxicology, to crop and stress tolerance improvement in plants [248].

### Metabolomics as an Investigatory Tool in Abiotic Stress Responses and Defence Priming

Metabolomics is a multidisciplinary ‘*omics*’ science that has proven indispensable in interrogating cellular biochemistry and metabolism and has established itself as a powerful research tool to address biological questions related to plant-environment interactions [249,250,251]. The plant kingdom is reported to contain a diverse array of between 200,000 and 1,000,000 metabolites which vary in class, chemical structure and polarity [247,252]. Owing to this wide chemical diversity, as well as the wide concentration range, there is no single analytical platform currently for the comprehensive examination of the entire metabolome *in toto* [253]. Consequently, a combination of different analytical platforms is often employed in plant metabolomics to detect and characterise these diverse compounds as holistically as possible. Current plant metabolomics approaches are reliant on either mass spectrometry (MS) or nuclear magnetic resonance (NMR) based approaches. Several comprehensive protocols on these approaches have been published [254,255], along with several excellent reviews [256,257,258,259]. Additionally, MS systems are often coupled to chromatographic platforms such as gas chromatography—mass spectrometry (GC-MS) and liquid chromatography—mass spectrometry (LC-MS); and such analytical systems have become popular in metabolomics studies, providing more sensitive detection of metabolites and wide coverage of the metabolome under consideration. 

Metabolite profiling of plants growing under abiotic stress conditions has provided crucial information about changes at biochemical and molecular levels underlying plant growth and adaptation. It is worth mentioning that metabolomic changes that have been reported in plants subjected to stress conditions are dependent on different causes; thus, these metabolic alterations have different significance and are expected to differently correlate with stress tolerance. Metabolomic reprogramming due to adverse environmental conditions involves complex and highly regulated molecular events some of which include (1) the stability and catalytic activity of enzymes involved in the biosynthesis/degradation of particular metabolites, (2) the adjustment of metabolite concentrations to re-establish homeostasis and normal metabolic fluxes and (3) the accumulation of compounds involved in mediating stress tolerance mechanisms [260].

Under abiotic stress conditions, the total number, concentration and types of metabolites are significantly altered. The alteration in gene expression is directly reflected in the metabolite profiles of plants. Acquiring knowledge about these differential metabolite profiles (which play a vital role in the growth, development and survival of the plant), and their modulation upon the onset of various abiotic stresses is highly fundamental. Reported metabolite changes have opened up the scope for the identification of viable metabolic markers which are important for abiotic stress tolerance of plants [247,261,262,263]. Numerous studies have reported on the use of metabolomics to study metabolite fluctuations in plants under stressful conditions [264,265,266,267,268,269]. Thus, metabolomics has become an indispensable tool in comprehending molecular mechanisms underlying abiotic stress responses. 

For example, upon exposure to abiotic stresses such as temperature, salinity and drought, plants accumulate a wide of compatible osmolytes which primarily function to maintain turgor. The accumulated solutes which vary among species include sugars (glucose, sucrose and fructose), polyols, betaines and amino acids such as proline [82,270]. Some of these compounds are known to play roles as osmoprotectants, low molecular weight chaperones, photosystem II complex stabilisers and ROS scavengers [271]. Additionally, some metabolites may act as chelating agents (sequestering toxic metals and ions), energy sources and signalling molecules under abiotic stress [271,272]. Metabolite fluctuations in response to individual abiotic stresses such as drought, salinity or heat have been widely studied and comprehensive reviews on this topic can be found in the literature cited herein [247,273,274]. The accumulation of various metabolites is one of the key mechanisms that plants use to cope with abiotic stresses; however, these natural mechanisms are not always adequate to ensure plant survival in all abiotic stress conditions; hence, the exploration of plant priming for more rapid and robust defence responses. As previously mentioned, priming mechanisms are described at different levels ranging from the epigenome to the metabolome, however, the metabolome is largely unexplored. Additionally, deciphering of the priming event which leads to intense and faster defence responses is far from being fully fathomed at biochemical and molecular levels, consequently, dissecting metabolomic changes induced by priming may provide insight into some of the key underlying priming mechanisms.

Evidence on plant metabolic changes that occur as a result of priming have been reported and include the reprogramming of the primary metabolism and differential biosynthesis of secondary metabolites [171,275,276,277,278], which are stored in a form of ‘metabolic memory’ or ‘metabolic imprint’, resulting in rapid and robust defence responses upon subsequent challenges [174,279]. For example, the accumulation of sugars, amino acids and hormones or their conjugates as key metabolic events during the priming phase has been reported to render the plant in a state of alertness upon subsequent environmental challenges [165,280]. Additionally, a study by Pastor et al. [3] reported on metabolic changes that take place during the priming phase. Following chemical priming by BABA to determine if priming pre-conditions the plant for attack by activating relevant metabolic pathways, *A. thaliana*’s primary metabolism was found to be boosted through alterations of the tricarboxylic acids (TCA) namely citrate, fumarate, malate and oxoglutarate. Furthermore, the amplification of the phenylpropanoid biosynthesis and the octadecanoic pathway was also observed. 

The metabolic reprogramming of the primary and secondary metabolism indicates that multiple metabolic pathways are involved in the priming phenomenon. The interconnectedness of these metabolic pathways has been reported to have feedback loops that allow for rapid activation of cellular defences to potential adverse conditions such as abiotic stress [169]. Despite the exponentially increasing efforts to elucidate these metabolic changes, gaps still exist in understanding the comprehensive molecular and biochemical mechanisms involved in the priming phenomenon due to its complexity. Regardless of these limitations, defence priming is unquestionably one of the key adaptive mechanisms plants employ under constantly fluctuating environmental conditions. Two approaches exist for investigating the metabolome: untargeted—and targeted analysis, with the typical workflow comprising of three main experimental steps namely sample preparation, data acquisition and data mining [27,281,282,283]. An untargeted analysis is used for discovery-driven studies such as the depiction of metabolomic changes induced by specific treatments, disease or genetic changes [284], simultaneously measuring as many metabolites as possible in the system in an unbiased manner. By contrast, a targeted analysis aims at identifying and quantifying metabolites in selected biochemical pathways, or specific classes of compounds in a hypothesis-driven approach [285].

As highlighted in sections above, the priming phenomenology is described at different layers including the metabolome and epigenome which can be explorable avenues for the underlying priming effect of biostimulants to enhance plant protective machinery against environmental stresses. Plant priming, therefore, presents a promising alternative approach due to the long-term and broad-spectrum resistance it provides against abiotic stress, providing an effective mechanism for crop protection under abiotic stresses. Accordingly, a comprehensive and mechanistic understanding (at molecular and cellular levels) of the beneficial effects of the biostimulants involved in priming could pave the way to design novel strategies that will aid plants in adverse environmental conditions, thus contributing to sustainable food security. Biostimulants, such as plant growth-promoting rhizobacteria (PGPR)-based formulations, therefore represent potentials to provide sustainable and economically favourable solutions that could introduce novel approaches to improve agricultural practices and crop productivity. However, to effectively establish and devise novel biostimulant-based agricultural strategies, there is a necessity to firstly understand the physiology and biochemistry governing the interactions between biostimulants and plants at both cellular and molecular levels. This knowledge gap—decoding molecular and physiological mechanisms underlying biostimulant action (by exploring the epigenomic and metabolomic approaches)—is one of the main bottlenecks that hamper the biostimulant field and industries from implementing and maximising the value of (traditional and novel) such formulations in agronomic practices. 

## 6. Conclusions and Perspectives

Climate change is depleting natural resources and exerting negative impacts on crop production, thus threatening food security. Modern agriculture is shifting toward improved crop yield and quality by exploiting the natural priming phenomenon in plants. In this context, the exploitation of biostimulants to enhance abiotic stress tolerance has increasingly become a strategy worth pursuing among the scientific community and agriculture industry. Biostimulants could pose as feasible alternatives in enhancing plant growth and increased stress tolerance. Numerous reports have elucidated the potential effects of biostimulant applications using various microorganisms such as PGPR; however, their comprehensive modes of action and underlying molecular mechanisms in relation to their priming effects and growth promotion are still enigmatic. This review discusses the growing literature and the current knowledge on plant defence responses to abiotic stresses and the integration of biostimulants in agricultural production systems, pointing out some knowledge gaps. The increasing use of biostimulants has demonstrated the positive effects of these formulations which include the modification of plant physiological and biochemical processes to enhance stress tolerance and productivity. This review further briefly highlights the application of metabolomics and epigenetic approaches in studying plant defence responses to abiotic stresses and defence priming.

## Figures and Tables

**Table 1:** Summary of different biostimulants and their abiotic stress-alleviating effect in plants.

Biostimulant	Crop	Stress Tolerance	Reference
*Azospirillum brasilense*	*Triticum aestivum*	Drought tolerance	[100]
*Azotobacter chrococcum*	*Zea mays*	Salt tolerance	[101]
*Azotobacter chrococcum*	*Triticum aestivum*	Temperature tolerance	[102]
*Azospirillum lipoferum*	*Triticum aestivum*	Salt tolerance	[103]
*Ascophyllum nodosum*	*Kappaphycus alvarezii*	Cold tolerance	[104]
*Ascophyllum nodosum*	*Camellia sinensis*	Drought tolerance	[105]
*Burkholderia phytofirmans*	*Vitis vinifera*	Cold tolerance	[106]
*Flavobacterium glaciei*	*Solanum lycopersicum*	Cold tolerance	[107]
*Pantoea dispersa*	*Triticum aestivum*	Cold tolerance	[108]
Fulvic and humic acids	*Festuca arundinacea*	Drought tolerance	[109]
Fulvic and humic acids	*Agrostis palustris*	Drought tolerance	[110]
Humic acid and phosphorous	*Capsicum annuum*	Salt tolerance	[111]
Humic acids	*Oryza sativa*	Oxidative and drought stress	[112]
Humic acids	*Phaseolus vulgaris*	Salt tolerance	[113]
Protein hydrolysates	*Zea mays*	Salt tolerance	[114]
Protein hydrolysates	*Lactuca sativa*	Salt tolerance, cold tolerance	[94,115]
*Ascophyllum nodosum*	*Arabidopsis thaliana*	Cold tolerance	[116]
*Ascophyllum nodosum*	*Agrostis stolonifera*	Heat tolerance	[117]
*Ascophyllum nodosum*	*Spinach oleracea*	Drought tolerance	[118]
*Ascophyllum nodosum*	*Zea mays*	Cold tolerance	[119]

**Table 2:** Plant growth-promoting mechanisms induced by rhizobacteria.

Strain	Mechanism	Plant	References
*Enterobacter aerogenes* (LJL-5), *Pseudomonas aeruginosa* (LJL-13)	1-aminocyclopropane-1-carboxylic acid (ACC) deaminase	Alfalfa	[131]
*Burkholderia* sp. MTCC 12259	IAA, ACC deaminase	Rice	[132]
*Bacillus aryabhattai* MCC3374	ACC, IAA, N_2_ fixation, siderophore	Rice	[133]
*Streptomyces* sp. VITMS22	IAA	Mustard	[134]
*Azotobacter chroococcum* CAZ3	IAA, siderophores, ammonia and ACC deaminase	Maize	[135]
*Enterobacter* sp.	ACC deaminase, IAA, siderophore, N_2_ fixation	Rice	[136]
*E. aerogenes* MCC 3092	IAA production, ACC deaminase, nitrogen fixation and P solubilisation	Rice	[137]
*Bacillus safensis*	IAA, ACC deaminase	Wheat	[138]
*Enterobacter cloacae* HSNJ4	IAA	*Brassica napus* L. (rapeseed)	[139]
*Acinetobacter* strain RSC7	IAA	*Vigna radiate* (mung bean)	[140]
*Enterobacter ludwigii* PS1	Auxin, siderophore, Hydrogen cyanide	Sea buckthorn	[141]

**Table 3:** Epigenetic mechanisms induced in different crop species under abiotic stress.

Crop	Abiotic Stress	Epigenetic Mechanism	References
*Arabidopsis thaliana*	Salt and drought stress	Histone acetylation	[183]
*Arabidopsis thaliana*	High salinity stress	Histone acetylation	[184]
*Arabidopsis thaliana*	Cold stress	Hypermethylation	[185]
*Arabidopsis thaliana*	Salinity	Hypomethylation	[186]
*Hordeum vulgare*	Terminal drought stress	Hypermethylation	[187]
*Beta vulgaris*	Salt stress	Histone acetylation	[188]
*Hydrilla verticillata*	Metal (copper) stress	Hypermethylation	[189]
*Zea mays*	Heat	Histone acetylation	[190]
*Zea mays*	Cold	Hypomethylation	[191]
*Zea mays*	Cold	Histone acetylation	[192]
*Zea mays*	Cold	DNA demethylation	[193]
*Populas*	Drought stress	Hypermethylation	[194]
*Oryza sativa*	Salt stress	Demethylation,	[195]
*Vicia faba*	Drought stress	Demethylation	[196]
*Triticum aestivum*	Salt stress	Hypermethylation	[197]
